# Seizure Detection Devices

**DOI:** 10.3390/jcm14030863

**Published:** 2025-01-28

**Authors:** Christoph Baumgartner, Jakob Baumgartner, Clemens Lang, Tamara Lisy, Johannes P. Koren

**Affiliations:** 1 Department of Neurology, Clinic Hietzing, 1130 Vienna, Austria; c.lang@gesundheitsverbund.at (C.L.); johannes.koren@gesundheitsverbund.at (J.P.K.); 2 Karl Landsteiner Institute for Clinical Epilepsy Research and Cognitive Neurology, 1130 Vienna, Austria; jakob.baumgartner@gmx.at (J.B.); tamara.lisy@extern.gesundheitsverbund.at (T.L.); 3 Medical Faculty, Sigmund Freud University, 1020 Vienna, Austria

**Keywords:** seizures, automated detection, epilepsy, multimodal

## Abstract

Goals of automated detection of epileptic seizures using wearable devices include objective documentation of seizures, prevention of sudden unexpected death in epilepsy (SUDEP) and seizure-related injuries, obviating both the unpredictability of seizures and potential social embarrassment, and finally to develop seizure-triggered on-demand therapies. Automated seizure detection devices are based on the analysis of EEG signals (scalp-EEG, subcutaneous EEG and intracranial EEG), of motor manifestations of seizures (surface EMG, accelerometry), and of physiologic autonomic changes caused by seizures (heart and respiration rate, oxygen saturation, sweat secretion, body temperature). While the detection of generalized tonic-clonic and of focal to bilateral tonic-clonic seizures can be achieved with high sensitivity and low false alarm rates, the detection of focal seizures is still suboptimal, especially in the everyday ambulatory setting. Multimodal seizure detection devices in general provide better performance than devices based on single measurement parameters. Long-term use of seizure detection devices in home environments helps to improve the accuracy of seizure diaries and to reduce seizure-related injuries, while evidence for prevention of SUDEP is still lacking. Automated seizure detection devices are generally well accepted by patients and caregivers.

## 1. Introduction

Epilepsy is a disorder of the brain characterized by an enduring predisposition to generate epileptic seizures, including the neurobiological, cognitive, psychological and social consequences of this condition [1]. Epilepsy consists of a broad group of disorders/diseases with diverse etiologies, diverse electroclinical presentations and considerable variability in clinical outcomes [2]. With a prevalence of 0.8%, epilepsy represents one the most frequent neurological disorders, affecting 60 million individuals worldwide [3]. Approximately 30% of patients suffer from medically refractory epilepsy, i.e., they continue to experience seizures despite adequate and optimized treatment with antiseizure medications [4,5,6]. Medically refractory epilepsy is associated with increased risks of comorbidity, including psychiatric disorders, cognitive impairment, somatic disorders, injuries and mortality. Therefore, this results in a significantly impaired quality of life both in affected individuals and their caregivers [7,8]. Epilepsy thus poses a significant burden both on the affected individuals and on their families, but also on society. A major impact of epilepsy arises from the lack of objective documentation of seizures and epilepsy severity and from the unpredictable occurrence of seizures [9,10,11,12,13].

Reliable automated seizure detection devices could significantly reduce the impact and burden of epilepsy for several reasons.

First, objective documentation of seizure frequency, seizure type and seizure severity represents the most important target parameter both for epilepsy treatment in an everyday clinical setting, and for the evaluation of medical and non-medical interventions in clinical trials. However, seizure documentation by the affected individuals, their relatives and caregivers is highly inaccurate and unreliable. In a video-EEG monitoring study, 55.5% of all seizures, 73.2% of focal impaired awareness seizures, 26.2% of focal aware seizures, 41.7% of bilateral tonic-clonic seizures, 85.8% of seizures occurring out of sleep and 32.0% of seizures occurring out of wakefulness were not documented by the patients [14,15]. Similar results could be obtained in a study using ultra-long-term EEG recordings with intracranial electrodes, where only a poor correlation between seizures reported by patients and seizures objectively documented on an intracranial EEG could be observed [16]. This seizure detection problem can therefore be regarded as a major problems in clinical epileptology [15,17]. Reliable automated seizure detection devices could help to solve this seizure documentation problem and thus provide an important biomarker for treatment evaluation in clinical epileptology.

Second, persons with epilepsy suffer from a 24–34 fold increased risk of dying suddenly and unexpectedly from sudden death [18,19,20]. Sudden unexpected death in epilepsy (SUDEP) represents the most frequent cause of sudden death in persons with epilepsy, especially in children and younger adults [18,20]. The incidence of SUDEP ranges from 0.9–2.3 per 1000 person-years in the general epilepsy population to 1.1–5.9 in patients with medically refractory epilepsy [18,20]. SUDEP causes 10–50% of deaths in patients with chronic epilepsies [18,20]. Focal to bilateral tonic-clonic and generalized tonic-clonic seizures represent a major risk factor for SUDEP, increasing the risk by a factor of 30; nocturnal focal to bilateral tonic-clonic and generalized tonic-clonic seizures in persons sleeping alone and unsupervised increase the risk by a factor of 67 [18,19,20,21]. Automated seizure detection devices instantly identifying and alerting family members or caregivers to focal to bilateral tonic-clonic and generalized tonic-clonic seizures therefore could help to prevent SUDEP, although evidence for this currently is still lacking [10,13,22].

Third, persons with epilepsy are at an increased risk for seizure-related accidents and injuries [23,24,25]. Specifically, 60% of patients with bilateral tonic-clonic and generalized tonic-clonic seizures suffer from an injury associated with a tonic-clonic seizure within one year, and 25% from a serious injury [26]. Automated seizure detection and warning devices could potentially be helpful to reduce seizure-related injuries.

Fourth, the unpredictability of seizures with potential social embarrassment represents one of the major concerns of people with epilepsy and contributes to the stigma of epilepsy [27,28,29,30]. Automated seizure detection and warning devices could thus help to reduce the stigma of epilepsy.

Finally, automated seizure detection devices could pave the way to seizure-triggered on-demand epilepsy treatment suppressing ongoing seizures or even preventing seizure generation [17,31,32,33,34,35].

Several large surveys among persons with epilepsy, their relatives, their caregivers and healthcare professionals documented that there is an urgent need for wearable seizure detection devices and that there is a high degree of willingness to wear such devices long-term in the everyday environment [36,37,38,39,40,41,42,43].

## 2. Performance and Measurement Parameters Used for Automated Seizure Detection Devices

Performance of seizure detection devices is usually based on detection sensitivity, false alarm rate (FAR) and detection delay or latency. While true positives (TP) refer to seizures correctly detected by the algorithm, false negatives (FN) refer to seizures missed by the algorithm, and false positives or FAR refer to seizure detections by the algorithm when no seizure actually occurred. Sensitivity is defined as the ratio of true positives TP/(TP + FN). Detection delay or latency is defined as the time interval between the time of seizure onset and the time when the automated algorithm sets the alarm [31,44,45,46]

Measurement parameters used in seizure detection devices include EEG as well as motor and autonomic correlates of seizures [11,47].

Ictal EEG changes measured on scalp-EEG, subcutaneous EEG and intracranial EEG as the electrophysiological correlates of epileptic seizures should represent the most sensitive and specific parameters for seizure detection [11,47].

Many seizures types, especially generalized tonic-clonic and bilateral tonic-clonic seizures, but also generalized tonic seizures and focal hyperkinetic (hypermotor) seizures are characterized by prominent motor manifestations, which can be detected by surface electromyography (sEMG) and accelerometry (ACC) [11,47].

Finally, epileptic seizures frequently cause an activation of the central autonomic network comprising the insular cortex, the cingulate cortex, the hypothalamus and the amygdala, resulting in changes of various autonomic parameters, including heart rate, respiration rate, oxygen saturation, skin temperature and sweat secretion [48,49,50]. Heart rate can be measured by electrocardiography (ECG) and photoplethysmography (PPG), respiration rate by respiratory monitors, oxygen saturation by pulse oximetry, skin temperature by surface temperature sensors and sweat secretion by electrodermal activity (EDA) [11,47]

## 3. Non-Invasive EEG-Based Seizure Detection

### 3.1. Scalp-EEG Using the 10–20 Electrode System and the Standardized EEG Electrode Array

Scalp-EEG-based seizure detection devices using the classical 10–20 electrode system or the standardized EEG electrode array of the International Federation of Clinical Neurophysiology (IFCN) [51] have been investigated in the setting of inpatient video-EEG-monitoring and provide sensitivities of 75–90% with false alarm rates (FAR) of 0.1–5 per hour [11,31,46,52,53]. EEG seizure patterns with short duration, low amplitude, circumscribed focal activity, high frequency and unusual morphology are difficult to detect, as well as EEG seizure patterns obscured by artifacts. Conversely, FARs are mainly caused by physiological and pathological interictal EEG activities as well as by various artifacts [11,31,46,52,53]. Therefore, detection algorithms generally perform better in seizures of temporal lobe origin than in seizures of extratemporal origin [54,55].

Comparisons of commercially available seizure detection software packages, including BESA (v2.0) (BESA Epilepsy, Gräfelfing, Germany), Encevis (v1.9.2) (AIT, Austrian Institute of Technology, Seibersdorf, Austria) and Persyst (v14) (Persyst Development Corporation, Solana Beach, CA, USA) yielded sensitivities of 67.6–81.0%, FARs of 0.2–0.9 per hour and detection delays of 20–30 s for earlier versions [53]. Performance was better for more recent versions, with sensitivities of 84–93% and FARs of 1.7–5.5 per 24 h [56]. However, wearing a full set of scalp EEG electrodes for prolonged time periods is evidently not acceptable for patients, and is therefore not feasible in an outpatient setting [11]. Therefore, several mobile devices for long-term EEG monitoring in an ambulatory setting have been developed.

Biondi et al. [57] performed a systematic review on non-invasive mobile EEG, including 21 different studies from 23 full-text articles and 6 abstracts and 14 device types. According to this review, automated seizure detection was investigated in four studies [58,59,60,61]. In patients with typical absences, sensitivities of 98% with FARs of 0.23–0.91 per hour could be obtained [58,59]. In patients with focal seizures, sensitivities of 90% with FARs of 0.087 per hour were observed [60]. Finally, a status epilepticus alert automatically analyzing seizure burden achieved a sensitivity of 100% and a specificity of 93% [61].

Ulate-Campos and Loddenkemper [62] reviewed automated seizure detection from a non-invasive limited-lead EEG, including nine articles [58,59,60,63,64,65,66,67,68]. Three articles [58,59,60] were already included in the review of Biondi et al. [57] and are mentioned above. In five studies on patients with focal epilepsies using behind the ear electrodes, sensitivities of 52.0–96.3% and FARs of 0.02–7.1 per hour were reported [63,64,65,66,67]. Performance was better in temporal than in extratemporal seizures [66,67], and deteriorated significantly in an outpatient setting compared to an inpatient setting in the epilepsy monitoring unit [67]. Personalized seizure detection algorithms improved performance [64,65]. For the detection of typical absences, the mean sensitivity was 78.8%, median sensitivity 92.9% and FAR 0.5 per hour [68].

In the following section, some selected studies will be reviewed.

### 3.2. Ear Electrodes

Simultaneous ear-EEG and scalp-EEG showed no differences in sensitivity or specificity for seizure detection in 15 patients with temporal lobe epilepsy [69]. A novel, discreet, unobstructive in-ear EEG system achieved a sensitivity of 86.4% with a FAR of 0.1 per day; the median recording time was 13 h, and the electrodes were well tolerated [70]. A limitation of ear electrodes in comparison to other devices is that only two electrodes per earbud (one placed in the cymba conchae and the other in the ear whole canal) are available, facilitating mainly recordings from the basal and lateral temporal lobe [70].

### 3.3. Behind-the-Ear Electrodes

Gu et al. [63] compared behind-the-ear EEG with scalp EEG in 12 patients with focal epilepsies. Automated seizure detection using a support vector machine (SVM) algorithm yielded similar results for scalp EEG (median sensitivity 100%, FAR 1.14 per hour) and for behind-the-ear EEG (median sensitivity 94.5%, FAR 0.52 per hour).

Vandecasteele et al. [66] compared visual and automated seizure detection of behind-the-ear EEG in 54 patients with focal epilepsies. Visual analysis of the behind-the-ear electrode recordings showed a sensitivity of 65.7% with a specificity of 94.4%, patient-independent automated seizure detection yielded a sensitivity of 64.1% with a FAR of 2.8 per 24 h and patient-specific automated seizure detection achieved a sensitivity of 69.1% with a FAR of 0.49 per 24 h.

Carvalho et al. [71] successfully applied a wearable two-bipolar-channel EEG system (Neury-2) facilitating 24 h recordings for repeated spike index (SI) quantification in 38 patients with continuous spike-wave of sleep (CSWS) syndrome, which is clinically relevant for accurate and objective monitoring of disease activity and treatment response.

Swinnen et al. [59] applied a two-channel behind-the-ear EEG system (Sensor Dot (SD)) with a patient-specific absence seizure detection algorithm in 12 patients with typical absence seizures. The automated detection algorithm achieved a sensitivity of 91.83% with a FAR of 0.91 per hour. Blind reading of the full sensor dot data set yielded a sensitivity of 81%, a precision of 0.89 and a F1 score of 0.73, while review of algorithm-labeled files led to a sensitivity of 83%, a precision of 0.89 and a F1 score of 0.87. Finally, patient self-reporting was associated with a sensitivity of 8%, a precision of 1.00 and an F1 score of 0.15. Notably, the time to review a 24 h recording was reduced from 1–2 h to 5–10 min using the automated detection algorithm.

You et al. [64] subjected behind-the-ear EEG recordings from 12 patients with various types of epilepsy to an automated seizure detection algorithm with a generative adversarial network (GAN) trained by unsupervised learning. An area under the receiver operating curve of 0.939 and a sensitivity of 96.3% with a FAR of 0.14 per hour could be achieved.

You et al. [65] subsequently developed a personalized deep-learning-based anomaly detection algorithm and applied it to behind-the-ear EEG recordings from 16 patients with epilepsy. While a sensitivity of 90.4% with a FAR of 0.83 per hour could be achieved without personal calibration, a sensitivity of 94.2% with a FAR of 0.29 per hour could be achieved with personal calibration.

Macea et al. [67] performed two-channel behind-the-ear EEG recordings (Sensor Dot (SD)) with an automated seizure detection algorithm during presurgical evaluation and at home in patients with focal epilepsy. In 16 inpatients, focal seizure detection sensitivity was 52%, FAR was 7.13 per hour, positive predictive value was 0.11 and F1 score was 0.002. In the 16 outpatients who were recorded for up to 8 months, sensitivity was 23%, FAR was 7.77 per hour, positive predictive value was 0.04 and F1 score was 0.001. Notably, 19 seizures during sleep not reported by patients as well as 2 auras were detected by the algorithm. Poor performance in the outpatient setting was attributed to artifacts and low signal quality.

Lehnen et al. [72] investigated a behind-the-ear seizure detection device called brain seizure detection (BrainSD) using four scalp electrodes in 32 patients with focal epilepsy. Average recording time was 41 h. Detection sensitivity was 78.6%, and FAR was not specified.

### 3.4. Epilog Sensors

Frankel et al. [60] developed miniature, wireless, wearable Epilog sensors recording a single EEG channel through a differential electrode pair spaced 18 mm center-to-center. Four Epilog sensors were placed bilaterally below the hairline approximately at the F7/F8 and P7/P8 locations, respectively, of the International 10–20 system. EEG data were converted into a 10 channel Remote EEG Monitoring platform (REMI) montage consisting of four individual sensor recordings and six sensor-to-sensor differential channels. The system was studied in 10 subjects with 24 focal onset seizures and 10 subjects without seizures and without epileptiform discharges on interictal EEG during video-EEG monitoring. The algorithm detected patients with seizures with a sensitivity of 100% and a specificity of 70%. Focal onset seizures were detected with a sensitivity of 90% with a FAR of 0.087 per hour.

In a subsequent study, Frankel et al. [73] studied detection of different seizure types in 75 seizures from 22 patients during video-EEG monitoring. In total, 71% of all seizures, 87% of electrographically generalized seizures and 56% of electrographically focal seizures could be correctly identified visually on Epilog recordings.

### 3.5. Headband Electrodes

Japaridze et al. [68] performed a prospective, multicenter, blinded phase three clinical trial using a one-channel EEG recorded with dry electrodes embedded into a wearable headband connected to a smartphone in 102 patients (median age = 10 years) with absence seizures. Average sensitivity per patient was 78.8%, median sensitivity was 92.9% and average FAR was 0.53 per hour. In 64.7% of all patients, no false alarms occurred. Furthermore, automated behavioral testing was performed in 36 seizures, which correctly documented non-responsiveness during 30 absence seizures and responsiveness during six absence seizures.

Nordli et al. [74] compared headband EEG with conventional EEG in 10 patients with absence epilepsy, and found concordant results in 80% of patients.

### 3.6. Non-Invasive EEG in Combination with Other Modalities

For the detection of focal seizures, adding a single electrocardiography (ECG) channel to a three-channel behind-the-ear EEG and temporal EEG increased sensitivity by 8–11%; i.e., from 82 to 90% and 81 to 92%, respectively, with an unchanged FAR [75].

For the detection of various seizure types, including focal impaired awareness seizures, focal aware seizures, focal to bilateral tonic-clonic seizures and seizures of unclear classification, adding an ECG to a behind-the-ear EEG increased sensitivity from 59.1 to 62.2% and reduced FAR from 6.5 to 2.4 per 24 h [76].

A two-channel behind-the-ear EEG was combined with electromyography (EMG), ECG, accelerometry (ACC) and gyroscope for the detection of tonic-clonic seizures [77]. While an EEG with a sensitivity of 100% and a FAR of 10.3 per 24 h performed best among single modalities, the combination of EEG and EMG improved performance with a sensitivity of 97.7%, a FAR of 0.4 per 24 h, a precision of 43% and an F1-score of 59.7%. The best performance could be obtained by the combination of EEG, EMG and ACC with a sensitivity of 90.9%, a FAR of 0.1 per 24 h, a precision of 75.5% and an F1-score of 82.5% [77].

The combination of two-channel EEG behind-the-ear electrodes, accelerometry and gyroscope data using a machine learning algorithm was studied in 13 patients with absence epilepsy in an ambulatory setting [78]. Using only EEG data, the algorithm achieved a sensitivity of 87% with a FAR of 4.03 per hour and a precision of 0.11. Using all modalities, a sensitivity of 86% with a FAR of 2.29 per hour and a precision of 0.18 could be obtained. The incorporation of accelerometry and gyroscope data thus resulted in a significant reduction of the FAR, especially those caused by motion artifacts [78].

## 4. Subcutaneous EEG Based Seizure Detection

Subcutaneous EEG (sqEEG) offers the opportunity to perform minimally invasive ultra-long-term EEG recordings for several months in an outpatient setting [79,80,81,82,83,84,85].

In comparison to intracranial EEG, seizure detection on sqEEG showed a sensitivity of 97%, a specificity of 91%, and an accuracy of 93% [86]. In normal subjects, sqEEG showed good agreement with scalp-EEG both in quantitative (power spectral density and coherence analysis) and qualitative (blinded subjective scoring by neurophysiologists) analysis [87]. In epilepsy patients, sqEEG is very similar to scalp-EEG in both time and time-frequency domains given that the distance between the electrodes is small, which points to the importance of appropriate positioning of the subcutaneous electrodes [79]. The signal quality of sqEEG, including signal impedance and power spectrum, remains stable over several months [84]. SqEEG home monitoring for up to 3 months was well tolerated with no serious adverse device-related side effects and provided objective seizure counts, which often differed considerably from patient-reported seizure counts [80]. During ultra-long-term recordings, adherence is very high, with sqEEG data covering 73% to 78.4% of the time [80,85]. SqEEG proved useful to detect unrecognized and breakthrough seizures, resulting in adjustments of treatment plans [88,89].

The enormous amount of data acquired during ultra-long-term sqEEG recordings cannot be visually analyzed within a reasonable time in a clinical setting. Therefore, the data need to be subjected to automated seizure detection algorithms [81]. To solve this problem, a deep neural network algorithm for two-channel seizure detection was developed, achieving a sensitivity of 88.8% and a FAR of 12.9 per day [90]. Appling this algorithm to sqEEG, data reduction ratios of 99.6% in epilepsy patients and of 99.9% in a control group could be obtained. Detection sensitivity was 86% with a FAR 2.4 per 24 h [83].

In a real-world pilot study, sqEEG was investigated in five patients with medically refractory epilepsy first in the epilepsy monitoring unit and subsequently in an outpatient setting for 3–6 months [91]. In the epilepsy monitoring unit, visual analysis of sqEEG identified all seizures in all patients. A semiautomated algorithm (EpiSight) detected all seizures in only three patients, while in the other two patients, only some seizures or no seizure could be detected. EpiSight detection depended on the characteristics of the ictal EEG patterns. In the outpatient setting, visual and semiautomated detections completely agreed in those three patients with agreement in the epilepsy monitoring unit. In the other two patients, semiautomated seizure detection varied from 0 to 83%. sqEEG showed that patients noticed only 25% to 86.7% of their seizures; three patients noticed less than half of their seizures. sqEEG was useful to objectively assess the effect of treatment adjustments [91].

A consensus-based expert recommendation regarded patients with focal impaired awareness seizures without predominant motor manifestations and seizures with medium to high voltage EEG patterns as the most suitable for sqEEG recordings [92]. In a recent study from three epilepsy centers, four major indication categories—namely diagnostic, therapeutic, risk assessment, patient guidance and medicolegal—including 11 clinical questions for sqEEG were identified, and corresponding clinical examples were presented [93]. The diagnostic category included diagnosis of epilepsy; the presence of active epilepsy, particularly in cases with rare seizures; differentiation of epileptic vs. non-epileptic events; and under-/overreporting of seizures. The therapeutic category included monitoring of therapeutic decisions and awareness of subclinical ictal events. The risk assessment category included assessment of seizure clustering and cycles as well as assessment of seizure severity, e.g., nocturnal tonic-clonic seizures. The patient guidance category included improving seizure awareness and self-reporting as well as improving seizure awareness to improve compliance. Finally, the medicolegal category was directed on objective evidence for complete seizure control [93]. sqEEG could also represent a valuable option for ultra-long-term monitoring in patients with intellectual disabilities. Potential benefits and implementing barriers for this indication were identified in a recent workshop [94,95].

The disadvantages of currently available sqEEG devices are the limited spatial coverage due to the low channel number necessitating a reasonable hypothesis on where to place the electrodes, the necessity of a minimal invasive procedure for device implantation and low device-associated risks including infection, bleeding and soreness around the implantation site [81,96].

## 5. Intracranial EEG-Based Seizure Detection

The responsive neurostimulation system (RNS) offers the opportunity to perform ultra-long-term intracranial recordings for years from one or two chronically implanted subdural strip or depth electrodes with four electrode contacts each [97,98,99,100,101]. The system is programmed to detect patient-specific epileptiform activity (mostly interictal epileptiform discharges) and to deliver brief electrical stimulation pulses in response to detections to suppress seizures [98,102]. Default detection settings after implantation apply a line length detector to detect changes in frequency and power of the ongoing intracranial EEG [98]. Subsequently, detection and stimulation parameters need to be adjusted and optimized manually by the treating physicians for several months after implantation, resulting in a considerable amount of review time for intracranial EEG [103]. Overall interrater agreement for the identification of seizures on the RNS system was 79%, and was thus only moderate with a κ of 0.57 [104]. Therefore, the application of automated detection algorithms based on deep leaning models could significantly improve adjustments as well as performance of the RNS system, and could save significant amounts of reviewing time [103].

A one-dimensional convolutional neural network (1D-CNN) model using 90-s raw signals as model input achieved a maximum mean binary classification area-under-precision-recall curve (AUPRC) of 0.84 for seizures occurring following a period of chronic recording (scenario 1), and a maximum mean AUPRC of 0.80 for seizures occurring immediately following implantation (scenario 2) with a seizure onset time estimation error of 12 s. Near-maximum accuracies were obtained with a seed size of 10 in both scenarios. Classification failures were caused by ictal electro-decrements, brief seizures, ictal patterns restricted to a single-channel, highly concentrated interictal activity, changes in the sleep-wake cycle, and progressive changes of ictal EEG patterns [105].

With a 2D-CNN model applied on 90-s spectrograms, the F1 score varied—depending on model complexity—between 77.8% with ∼1.5 million parameters and 90.2% with ∼23.5 million parameters using 30 patients’ data for training. A seizure onset time estimation error was not provided. The trained model showed an 93.5% agreement with independent human experts [106].

A 2D-CNN and recurrent neural networks (RNNs) applied to 90-s spectrograms using ∼1.6 million parameters, an F1 score of 0.88 and a seizure onset time estimation error of 3.4 s could be obtained. This approach could achieve a cross-patient overall accuracy of up to 90% without the need to add data from the given patient and with relatively low complexity in terms of both the number of model parameters and training data. It could be shown that RNS device detections were less accurate and more delayed in non-responders, which underlines the importance of improving seizure detection accuracy in order to increase the therapeutic efficacy of RNS [103].

Limitations of intracranial seizure detection with the RNS system include limited spatial sampling restricted by two electrodes with four contacts, the invasiveness of the procedure requiring brain surgery for electrode placement, possible complications including infections, poor wound healing, focal neurological deficits, lead breaks and intrinsic device failure, high cost, non-availability in Europe, and its labor-intensiveness both for physicians, engineers and technicians [96,107].

## 6. Cardiac-Based Seizure Detection

Epileptic seizures are associated with autonomic changes affecting heart rate with ictal sinus tachycardia representing the most frequent ictal heart rate change [108]. Ictal tachycardia occurs in at least one seizure in 82% of patients and can be observed in 12% of subclinical seizures, in 71% of focal seizures, in 64% of generalized seizures and in 76% of mixed seizure types [108]. The average absolute heart rate increase is 34.2 beats per minute (bpm) per seizure and 33.5 bpm per patient, corresponding to a relative change of more than 50% when compared to baseline heart rates. Concerning the timing of ictal tachycardia, the majority of studies demonstrated a significant rise in heart rate within the first 30 s of seizure onset, indicating that ictal tachycardia is useful as a biomarker for seizure detection [108]. Hirsch et al. [109] studied the timing of ictal tachycardia in relation to clinical seizure semiology, scalp-EEG and invasive EEG in 78 seizures from 13 patients. While ictal tachycardia occurred after seizure onset on intracranial EEG in all seizures with a mean latency of 21.6–23.7 s, ictal tachycardia was observed before clinical seizure onset in 10 out of 13 patients and 56 out of 78 seizures with a mean latency of 6.5–9.5 s, and before seizure onset on scalp EEG in 9 out of 13 patients and 48 out of 78 seizures with a mean latency of 7.8–14.0 s. The authors concluded that ictal tachycardia represents an ictal rather than a preictal phenomenon [109].

Closed-loop cardiac-based seizure detection is used in the newer vagus nerve stimulator models (Aspire SR, SenTiva LivaNova, Houston, TX, USA). Heart rate (HR) is monitored, and electrical stimulation is delivered when HR increases by 20–70% within 10 s compared to the baseline defined as the average HR over the previous 3 min. For a threshold of 20% HR increase, VNS cardiac-based seizure detection showed a sensitivity of 43 to 52.3% and a FAR of 7.15–9 per hour, while for a threshold of 60% HR increase, a sensitivity of 8–13% and a FAR of 0.49–0.5 per hour could be observed [9,110,111]. Ictal tachycardia as a biomarker for seizure detection therefore is mainly limited by its low specificity because HR can be influenced by physical exercise, emotional stress, medication, age and sleep quality [9,112,113].

Heart rate variability (HRV) measuring reciprocal sympathetic and parasympathetic influences represents another parameter that can be used for seizure detection [112,113,114]. Specifically, the modified cardiac sympathetic index (CSI) showed a sudden increase in sympathetic activity around seizure-onset that exceeded 105% of the highest value during exercise and other HR increases without seizures, even when the absolute HR was greater than that during seizures [114].

Some earlier studies using HRV showed sensitivities of 77.0–96.4% with a FAR of 0.5–5.4 per hour [115,116,117,118].

Seth et al. [113] conducted a systematic review on cardiac-based seizure detection and prediction, including a total of 24 studies (20 studies on seizure detection and four studies on seizure prediction). Wrist-worn and chest-worn devices were most frequently used for data acquisition. The parameters used for seizure detection algorithms were HR (n = 11), HRV (n = 2) or a combination of HR and HRV (n = 6). Reported sensitivities ranged from 56 to 100% with a FAR of 0.02–8 per hour. Algorithms using HRV achieved slightly higher sensitivities than algorithms using HR only. While most studies were retrospective, data on real-time detection were limited. In the following section, some selected studies on cardiac-based seizure detection are summarized.

Jeppesen et al. [119] studied HRV for the detection of non-convulsive seizures. They included 126 seizures (108 non-convulsive and 18 convulsive) from 43 patients recorded during long-term video-EEG. Patients were defined as responders if >66% of their seizures were detected by the best-performing HRV algorithm. In total, 53.5% of patients were classified as responders. In these patients, detection sensitivity was 93.1% for all seizures and 90.5% for non-convulsive seizures. FAR was 1.0 per 24 h and 0.11 per night. Median seizure detection latency was 30 s. An ictal HR increase of >50 bpm identified responders with a positive predictive value of 87% and a negative predictive value of 90%.

In a subsequent study, Jeppesen et al. [120] recorded ECG using a wearable device (ePatch) and applied a seizure detection algorithm based on HRV using patient-specific cutoff values. Patients were defined as responders if ictal heart rate increased more than 50 bpm. A total of 11 out of 19 patients were classified as responders. In these patients, detection sensitivity was 87% for all seizures, 90% for convulsive seizures, 100% of focal impaired awareness seizures and 50% of focal aware seizures. Median sensitivity per patient was 100%, i.e., in nine patients, all seizures were detected. FAR was 0.9 per 24 h and 0.22 per night.

De Cooman et al. [121] proposed a personalized HR-based seizure detection algorithm trained a couple of days per patient. They studied 227 focal-impaired awareness seizures from 24 patients with temporal lobe epilepsy. Sensitivity was 71% with a FAR of 1.9 per hour, which corresponded to an average decrease in FAR of 37% compared to the patient-independent algorithm used as a reference.

Glasstetter et al. [122] studied the potential of wrist-worn photoplethysmography (PPG) for seizure detection, including its sensitivity to movement artifacts in 62 focal seizures from 28 patients with ictal tachycardia on ECG. Ictal tachycardia could be identified by PPG in 37 out of all 62 seizures (59.7%), 9 out of 19 seizures without motor manifestations (47.3%), 7 out of 8 seizures with motor manifestations not affecting the extremities (87.5%) and 21 out of 35 seizures with motor manifestations affecting the extremities (60%). Ictal tachycardia occurred prior to the onset of motor manifestations, affecting the extremities in 14 out of 21 seizures (66.7%). In 30 out of 37 seizures (81.1%), ictal tachycardia on PPG and ECG showed a good temporal agreement (<10 s) and occurred within 5.0 s of EEG onset.

Jahanbekam et al. [123] compared ECG-based seizure detection using an algorithm based on feature extraction and machine-learning between three patient groups, namely in patients undergoing video-EEG monitoring with additional devices (accelerometry, ECG, electrodermal activity; group 1), in patients undergoing VEEG with a one-lead ECG (group 2), and in patients undergoing mobile EEG recordings with a one-lead ECG (group 3). In group 1, which was used for the development of the algorithm, the F1 score was 56%, the sensitivity was 67%, the precision was 45% and the FAR was 0.7 per 24 h when ECG data were used alone; the addition of accelerometry and electrodermal activity did not improve performance. In group 2, similar results could be achieved (F1 score 51%, sensitivity 39%, precision 73% and FAR 0.4 per 24 h). However, in group 3, performance was significantly worse (F1 score 27%, sensitivity 31%, precision 23%, FAR 1.2 per 24 h). The authors pointed towards the importance of training and test data sets as well as the susceptibility of algorithms to different conditions, i.e., patients lying in bed during video-EEG monitoring versus freely moving around at home.

Hegarty-Craver et al. [124] evaluated cardiac- and accelerometer-based seizure detection in 25 seizures from 18 children. Based on ECG alone, 11 out of 12 generalized seizures with clonic or tonic activity and 7 out of 13 focal seizures without generalization could be detected with a FAR of one per day. The addition of accelerometer data did not improve performance, but shortened detection time for 4 of the 12 generalized seizures.

Jeppesen et al. [125] applied a subcutaneously implantable cardiac monitor with a HRV algorithm for seizure detection in six patients monitored during epilepsy monitoring unit recordings and at home for 1 to 8 months. In total, 50 of 54 focal seizures (sensitivity = 92.6%), 12 of 13 seizures in the epilepsy monitoring unit (sensitivity = 92.3%) and 38 of 41 seizures in the ambulatory setting (sensitivity = 92.7%) could be detected. FAR was 2.7 per 24 h.

Arbune et al. [126] correlated peri-ictal HRV changes with seizure duration, intensity of electromyography-documented ictal muscle activity, and the presence and duration of post-ictal generalized EEG suppression (PGES) with the question of their potential usefulness to measure seizure severity and to assess the risk of sudden unexpected death in epilepsy (SUDEP). They included 75 motor seizures from 40 patients. In the post-ictal period of all major motor seizures, HRV showed a significantly decreased parasympathetic and increased sympathetic activity, and was associated with longer PGES (>20 s), longer seizure duration and greater intensity of ictal muscle activity. Increased sympathetic activity was significantly higher for generalized tonic-clonic seizures (GTCS) than for non-GTCS. Furthermore, mean post-ictal heart rate independently predicted PGES duration, seizure duration and intensity of ictal muscle contraction.

## 7. Surface-EMG-Based Seizure Detection

Surface EMG (sEMG) facilitates the detection of seizures with prominent motor manifestations, especially of generalized and bilateral tonic-clonic seizures and of tonic seizures. sEMG recordings can be performed from various muscles, including the deltoid muscle, the biceps brachii muscle and the tibialis anterior muscle, with the biceps brachii muscle representing the most widely used muscle for seizure detection.

Several large studies reported sensitivities of 93.8–100%, FAR of 0.67–1.44 per 24 h and detection delays of 7.7–15.2 s for the detection of generalized and bilateral tonic-clonic seizures [127,128,129,130].

sEMG demonstrated that the tonic phase of generalized tonic-clonic seizures and tonic seizure were quantitatively different, i.e., that for the whole seizure period, the frequency values (relative power > 100 Hz) of tonic seizures were significantly higher than for the tonic phase of generalized tonic-clonic seizures, whereas amplitude was significantly higher for the tonic phase of generalized tonic-clonic seizures than for tonic seizures [131]. These results suggest different pathomechanisms with recruitment of more motor neurons, including high threshold motor neurons during tonic seizures and prominent synchronization among motor units during generalized tonic-clonic seizures [131]. Furthermore, sEMG allowed a detailed characterization of the temporal dynamics of generalized tonic-clonic seizures, demonstrating a high-frequency (64–265 Hz) component dominating during the tonic-maintenance phase, and a low-frequency component (2–8 Hz) with peaks during the onset phase and during the clonic phase (especially at the transition from the tonic to the clonic phase) representing inhibitory phenomena [131]. These findings suggest that the same inhibitory mechanisms trying to prevent the build-up of seizure activity at seizure onset eventually contribute to seizure cessation at seizure offset [131].

sEMG was also useful to automatically classify seizures with upper-extremity motor activity in tonic-clonic, tonic only, clonic only or other motor seizures at 72% accuracy [132].

Based on the analysis of EMG artifacts, sEMG could automatically differentiate epileptic from psychogenic non-epileptic convulsive seizures with an overall diagnostic accuracy of 95%, generalized tonic-clonic seizures were correctly classified at an accuracy of 96%, and psychogenic non-epileptic seizures at an accuracy of 95% [133].

## 8. Accelerometer-Based Seizure Detection

Accelerometers (ACCs) represent the most frequently used sensors to measure movements during epileptic seizures [134,135]. Almost invariably, three-dimensional ACCs are used for quantifying changes in motion in three orthogonal directions. Rhythmic clonic movements during tonic-clonic seizures result in a characteristic three-dimensional accelerometric ‘fingerprint’. ACCs are usually placed on the upper arm or on the wrist [134,135].

For the detection of tonic-clonic seizures ACC-based seizure detection yielded an average sensitivity of 93% (95% CI = 85–99%) and an overall FAR of 2.5 per 24 h (95% CI = 1.95–3.1) according to a systematic review and meta-analysis [136].

For the automated detection of tonic seizures with visible clinical manifestations using a wearable wristband movement sensor (ACC and gyroscope), a sensitivity of 100%, an average FAR of 0.16 per night and a mean detection latency of 14.1 s could be achieved [137].

Wrist-worn ACC-based devices could differentiate epileptic generalized tonic-clonic seizures from psychogenic convulsive psychogenic non-epileptic seizures [138,139]. Sensitivities for correct classification of convulsive psychogenic non-epileptic seizures were 95.45% to 100%, specificities for the correct classification of generalized tonic-clonic seizures were 72.7% to 94.87% [138,139].

Automated detection of post-ictal ACC silence correctly identified expert-labeled postictal patient immobility [140]. The duration of the post-ictal ACC silence was correlated with the duration of patient immobility, with age and with duration of post-ictal generalized electroencephalography suppression. Automatic detection of post-ictal ACC silence could therefore help to identify persons at an increased risk for SUDEP [140].

## 9. Electrodermal-Activity-Based Seizure Detection

Electrodermal activity (EDA) describes the change in the electrical properties of the skin and essentially corresponds to the state of eccrine sweat glands in the skin. Eccrine sweat glands are activated by sudomotor nerves, which are exclusively innervated by the sympathetic autonomic nervous system. Therefore, EDA represents an ideal measure for the measurement of sympathetic activity. EDA is composed of two main components: Tonic long-term EDA fluctuations or electrodermal level (EDL)-reflecting slow varying tonic sympathetic activity and phasic short-term EDA fluctuations or an electrodermal response (EDR) generated by fast varying phasic sympathetic activity elicited by different external or internal stimuli [141,142,143,144].

While the neural mechanisms underlying EDA are complex and still not fully understood [143,145], electrical stimulation of limbic structures, including the amygdala, the anterior and posterior hippocampi and the cingulate gyri in humans, caused an EDA increase that is more pronounced to ipsilateral stimulation [146]. Seizures cause an EDR as a result of epileptic activity involving the central autonomic network [48,143,147,148]. While the most pronounced EDRs can be obtained from the face, the palms and the soles, the wrists also show good EDR responses facilitating long-term outpatient measurements using wrist-worn devices for seizure detection [149].

Casanovas Ortega et al. [148] performed a systematic review and meta-analysis on EDA during seizures. They included 19 studies with 550 patients and 1115 seizures. In all studies, an increased ictal and post-ictal EDA could be observed. Pre-ictal EDA responses were reported in three studies, i.e., a pre-ictal EDA increase in two studies [150,151] and a pre-ictal EDA decrease in one study [152]. In the six studies included in the meta-analysis, an EDA increase was found in 82 out of 100 seizures [152,153,154,155,156]. The EDA response was higher and longer-lasting in focal to bilateral tonic-clonic seizures and generalized tonic-clonic seizures than in focal seizures. In focal motor seizures with impaired awareness, there is a characteristic time evolution of different bio-signals, with an increase in heart rate occurring first, followed by motor manifestations captured on ACC, and finally a sweat increase measured as the last-appearing EDA change [157,158]. The magnitude of the postictal EDA response correlated with the duration of the post-ictal generalized EEG suppression (PGES) [159,160]. EDA response as a measure of sympathetic activation and vagal suppression was more pronounced in children, while PGES was shorter in children than adults [159].

## 10. Multimodal Seizure Detection

Most modern seizure detection devices use a multimodal approach measuring several different parameters, including ECG, photoplethysmography (PPG), accelerometry (ACC), body temperature and electrodermal activity (EDA) [12,13,161]. For the detection of generalized tonic-clonic seizures, multimodal detection devices achieved sensitivities of 79.4–96% and FARs of 0.20–1.92 per 24 h (0–0.03 per night) with detection latencies of 13–29.3 s [12,130,154,162,163,164,165,166].

Seizure detection for different seizure types using wrist- and ankle-worn sensors measuring body temperature, EDA, ACC and PPG (blood volume pulse (BVP)) was studied with individually developed machine learning algorithms [157]. On the one hand, algorithms were trained and validated for seizure-type-specific detection of nine different seizure types, including focal to bilateral tonic-clonic seizures, generalized tonic-clonic seizures, focal tonic seizures, focal subclinical seizures, focal automatisms, focal behavior arrest, focal clonic seizures, generalized tonic seizures and generalized epileptic spasms (algorithm 1). On the other hand, a general seizure type-agnostic detection algorithm for all seizure types together (algorithm 2) was also developed. Algorithm 1 detected eight of the nine seizure types better than chance; only the detection of focal subclinical seizures was close to chance. Algorithm 2 performed better than chance for all nine seizure types, with the combination of ACC and BVP providing the best results. Thus, machine learning models trained on all seizures types together performed better than models trained on specific seizure types alone [157].

For the detection of tonic-clonic seizures, an Apple Watch with a research app continuously recording ACC and PPG signals yielded a sensitivity of 100%, a FAR of 0.05 per 24 h, a precision of 68% and a latency of 32.07 s in an epilepsy monitoring unit setting. In an ambulatory setting, sensitivity was 100%, FAR was 0.13 per 24 h, precision was 22% and latency was 37.38 s [167].

In a phase 2 study using an off-the-shelf digital watch, namely the Samsung watch (SM-R800), a machine learning algorithm provided a balanced accuracy of 0.93, a precision of 0.68, a sensitivity of 0.87 and a FAR of 0.21 per 24 h for the detection of generalized convulsive seizures in the epilepsy monitoring unit [168]. In patients without seizures, FAR was 0.28 per 24 h. Heart-rate-based features were superior to ACC/gyroscope-based features [168].

In an out-of-hospital study, 17 patients were monitored for up to 5 days using wearable ECG, ACC and behind-the-ear EEG. When training the algorithm on out-of-hospital recordings, the combined use of all modalities yielded a sensitivity of 100%, a FAR of 10 per 24 h and an F1 score of 0.75. When the algorithm was trained on the in-patient data and tested on the out-patient data, a sensitivity of 91%, a FAR of 18 per 24 h and an F1 score of 0.58 could be obtained using ECG and EEG data [169]. However, only 30.1% of the EEG data provided a usable signal [169].

In children, the detection of nightly major seizures defined as tonic-clonic, generalized tonic > 30 s, hyperkinetic or clusters (>30 min) of short myoclonic or tonic seizures using NightWatch, which records HR derived from PPG and movements by ACC, showed a sensitivity of 79.9% with a mean PPV of 26.7% and a FAR of 0.2 per hour [170]. When the alarm was only triggered when the patient was in the horizontal position, PPV improved to 55.5% and FAR decreased to 0.08 per hour, while sensitivity remained unchanged with 79.4% [170].

In a phase 4 study on children in the family home setting, NightWatch yielded a median detection sensitivity per participant of 100% and a median individual FAR of 0.04 per hour for the detection of major motor seizures (focal to bilateral or generalized tonic–clonic (TC) seizures, focal to bilateral or generalized tonic seizures lasting > 30 s, hyperkinetic seizures and a remainder category of focal to bilateral, generalized clonic, and ‘TC-like’ seizures) [171]. Furthermore, the caregiver’s stress decreased significantly [171].

In a pediatric population, seizure detection of a tonic-clonic seizure using the Empatica E4, measuring EDA, ACC and HR, yielded a sensitivity of 0.52 and FAR of 0.28 per 24 h across all ages [172]. Performance improved with patient age. Seizures under high antiseizure medication load or with shorter duration were detected worse. Clonic motor activity and peri/postictal increases in HR and EDA were the most informative parameters [172].

For the detection of tonic seizures in pediatric patients, including more than half with Lennox-Gastaut syndrome, the combination of behind-the-ear EEG, surface EMG, ECG and ACC/gyroscope yielded a sensitivity of 41%, a PPV of 9%, an F1 score of 14% and a median FAR of 0.75 per hour [173]. For nightly seizures lasting ≥ 10 s, sensitivity was 66%, PPV was 66% and F1 score was 66%; for nightly seizures lasting ≥ 20 s, sensitivity was 62%, PPV was 82% and F1 score was 71%, which was better than seizure diaries which reached a maximum sensitivity of 52% for nightly seizures lasting ≥ 20 s, even when caretakers slept in the same room [173].

## 11. Clinical Practice Guideline

The Working Group of the International League Against Epilepsy (ILAE) and the International Federation of Clinical Neurophysiology (IFCN) developed a clinical practice guideline on the use of wearable devices for automated seizure detection [10]. According to the methodology proposed by the ILAE Epilepsy Guidelines Working Group [174], the published evidence was reviewed and evaluated using The Preferred Reporting Items for Systematic Review and Meta-Analysis (PRISMA) statement [175], and recommendations were formulated following the Grading of Recommendations Assessment, Development and Evaluation (GRADE) system [176]. They found a high level of evidence for the accuracy of automated detection of generalized tonic-clonic seizures (GTCS) and focal-to-bilateral tonic-clonic seizures (FBTCS) based on three phase-3 studies using ACC [177], sEMG [129] and multimodal detection consisting of HR, PPG and 3D-ACC [163]. In these studies, a total of 880 seizures from 68 patients were included, and sensitivities of 90–96% and FARs of 0.2–0.7 per 24 h could be achieved [10]. The Working Group recommended the use of clinically validated wearable devices for automated detection of GTCS and FBTCS when significant safety concerns exist, especially in unsupervised patients who do not share a bedroom, but where alarms can result in rapid intervention (within 5 min) corresponding to a weak/conditional recommendation [10]. For the detection of seizures without a tonic-clonic component, only a moderate level of evidence was found based on eight Phase-2 studies applying EEG, PPG and ECG as detection modalities. A total of 1906 seizures from 152 patients were included, yielding sensitivities of 32–90% with FARs of 0.7–65 per 24 h [10]. The Working Group therefore did not recommend the clinical use of currently available wearable devices for seizure types other than GTCS and FBTCS as a weak/conditional recommendation.

The systematic review performed by the Working Group included papers published before 16 October 2019 [10]. In a recent review, the literature search was updated to include articles published between October 2019 and August 2023 [12]. Studies qualifying minimally as phase-two clinical validation trials according to the standards for testing and validation of seizure detection devices were selected [178]. In the updated systematic literature search, two phase-four studies on the performance of wearable seizure detection devices in a home setting were identified [171,179]. Overall satisfaction, perceived sensitivity and improvement in quality-of-life were significantly higher for validated devices [179]. Devices helped to reduce injuries related to tonic-clonic seizures [179]. Three additional phase-three studies reporting on the detection of tonic-clonic seizures and major motor seizures using ACC and EDA [165], ACC and HR [170] and an audio-video system [180] yielded sensitivities of 79.4–100% with FARs of 0.57–3.84 per day and detection delays of 7–37 s. A single phase-three study investigated the detection of absence seizures using a single-channel wearable EEG device [68]. Finally, 24 phase-two studies were found [12]. The authors concluded that the studies published after the clinical practice guideline only provide incremental knowledge and would not change the current recommendations [12].

Table 1 shows the advantages and disadvantages of various seizure detection devices. In Table 2, the use of various seizure detection devices in special patient populations is depicted. Table 3 shows seizure detection devices with a CE (Conformité Européenne) mark and/or with FDA approval. In Table 4, research gaps and their possible solutions in the future are summarized.

## 12. Conclusions

Automated seizure detection devices are based on the analysis of EEG signals recorded on scalp-EEG, subcutaneous EEG and intracranial EEG, on the analysis of motor manifestations of seizures measured with sEMG and ACC, and finally on the analysis of autonomic changes caused by seizures, including changes to heart and respiration rate, oxygen saturation, sweat secretion and body temperature [11,47]. Currently, generalized tonic-clonic and focal to bilateral tonic-clonic seizures can be detected with high sensitivity and low FARs [10,13]. On the contrary, reliable detection of focal seizures in the home environment is still suboptimal, and seizure detection devices are not generally recommended in this context [10,12,13]. Promising results have been obtained for the detection of absence seizures and automated testing of responsiveness during absence seizures in an ambulatory setting [68]. Different parameters can be optimal for detection of specific seizure types, and therefore different device types may be optimal for different patients. Personalized algorithms can improve the performance in individual patients [64,65]. Multimodal seizure detection devices in general provide better performance than devices based on single measurement parameters [76,77,78,157].

Automated seizure detection represents a means to objectively document seizure frequency, seizure type and seizure severity, which is highly unreliable based on currently used patient reports and diaries [14,15,16]. This would solve the seizure detection problem—one of the major problems in clinical epileptology [15,17]. Indeed, long-term use of seizure detection devices in home environments helped to improve the accuracy of seizure diaries in almost two-thirds of patients [179].

The fact that generalized tonic-clonic and focal to bilateral tonic-clonic seizures, especially during sleep, can be reliably detected by automated seizure detection devices is clinically relevant, because sudden unexpected death in epilepsy (SUDEP) only occurs in association with focal to bilateral tonic-clonic and generalized tonic-clonic seizures and mostly during sleep [18,20,21]. Nevertheless, evidence for SUDEP prevention by automated seizure detection devices currently is still lacking [10,13].

Automated seizure detection devices should help to prevent seizure-related accidents and injures. In an outpatient long-term study, automated seizure detection devices reduced the number of seizure-related injuries in 30% of patients [179].

Depending on whether seizure quantification or SUDEP and injury prevention are the goals, an individual off-line detection application or on-line detection, respectively, needs to be implemented [11,12].

Automated seizure detection devices are generally well accepted by patients and caregivers, and help to improve quality of life [179]. Nevertheless, major issues for all portable seizure detection devices remain, including comfort, ease of use and practicability in everyday life, which are critical for patient adherence.

Mobile seizure detection devices should be useful in patients with neurodegenerative diseases, especially in individuals with Alzheimer’s disease. Seizures are common in Alzheimer’s disease patients, but their detection can be challenging due to cognitive impairments and the potential for atypical seizure presentation. In Alzheimer’s disease, seizure detection devices would be useful for detecting seizures during sleep when the person is not observed, as well as for prevention of falls or injuries. Real-time seizure detection would allow timely intervention and better management of seizures by caregivers or family members. Finally, subtle seizures or even frequent interictal epileptiform discharges can accelerate cognitive decline in Alzheimer’s disease, which could be held back by prompt seizure recognition and treatment [181,182,183].

It should be noted that while the devices discussed in this review are useful for seizure detection, seizure prediction currently remains restricted to intracranial devices.

The performance of automated seizure detection devices for use intended in a clinical setting needs to be evaluated according to the proposed standards for testing and validation of seizure detection devices [178]. Finally, the applied algorithms should be transparent and interpretable [13].

## Figures and Tables

**Table 1:** Advantages and disadvantages of various seizure detection devices.

Device Type	Advantages	Disadvantages
ear electrodes	non-invasive; discreet; comfortable; easy to apply and remove; minimal motion artifacts; suitable for all-day home-monitoring	limited coverage especially for extratemporal regions; limited signal quality; problems for high-frequency detection and small-scale events; noise susceptibility
behind-the-ear electrodes	non-invasive; better signal quality than ear electrodes; easy to use; reduced motion artifacts; comfortable; less intrusive compared to headbands; aesthetic appeal; suitable for all-day home-monitoring	limited coverage especially for extratemporal regions; problems with noise and artifacts; requires high-quality dry or semi-dry electrodes; prolonged use may cause discomfort; visible depending on clothing
epilog sensors	non-invasive; high signal quality; covers extratemporal regions; discreet; comfortable; wireless and portable; small and lightweight; easy to use; suitable for all-day home monitoring	may require additional devices or apps for monitoring; can be sensitive to environmental noise; uncomfortable to wear for extended periods; costly
headband electrodes	non-invasive; lightweight; easy to wear; cost-effective; good coverage; lightweight and easy to wear; user-friendly; facilitates interactive monitoring; suitable for long-term monitoring; sensors monitoring other modalities can be integrated	limited coverage; lower signal quality (dry electrodes); bulkier and less discreet than other devices; can be uncomfortable; prone to movement artifacts; power and data storage limitations
subcutaneous EEG	minimally invasive; high sensitivity; low false alarm rates; reduced motion artifacts; stable signal quality over prolonged time periods; continuous monitoring; useful for treatment planning; comfortable; discreet; suitable for all-day home-monitoring	requires surgical procedure; limited coverage; risk of infection; device malfunctions; high cost; limited availability
invasive EEG	high sensitivity; low false alarm rates; highly localizing; minimal artifacts; stable signal quality over prolonged time periods; continuous monitoring; useful for treatment planning; discreet; suitable for all-day home-monitoring	invasive; complicated implantation; surgical risks; recovery time after implantation; high cost; limited availability
cardiac-based devices	non-invasive; easy to wear; comfortable; early seizure detection; short response time; real time alerts; possible seizure prediction; suitable for all-day home-monitoring	limited sensitivity—not specific for seizures; less sensitive for non-convulsive seizures; low specificity; interference from external factors (e.g., body movement, noise or device misplacement); problems in patients with heart conditions and in special populations (e.g., infants); complex data analysis
surface electromyography (sEMG)	non-invasive; high sensitivity and specificity for tonic-clonic seizures: comfort and convenience; affordable and accessible; continuous monitoring; real-time alerts; suitable for all-day home-monitoring	not sensitive to non-motor seizures; false alarms caused by other movements; battery life; requires correct electrode placement; skin contact issues; uncomfortable to wear for long periods; need for calibration
accelerometer (ACC)	non-invasive; small, lightweight and portable; easy to use; low power consumption; low cost; continuous monitoring; real-time alerts; suitable for all-day home-monitoring	not sensitive to non-motor seizures; false alarms caused by other movements; battery life
electrodermal activity (EDA)	non-invasive; early seizure detection; easy to use; comfortable; continuous monitoring; supplementary to other modalities; low power consumption; suitable for all-day home-monitoring	less specific for seizures compared to other methods; affected by stress, anxiety, physical activity and environmental factors like temperature; useful only for detecting seizures causing autonomic responses; need for calibration
multimodal devices	non-invasive; higher sensitivity and lower false alarm rates by combining data from multiple sensors; more robust to noise; comprehensive monitoring of different seizure types; suitable for all-day home-monitoring	higher power and battery consumption; higher cost; complex data processing; integration issues for combining different modalities; increased risk for false positive alarms

**Table 2:** Use of various seizure detection devices in special patient populations.

Patient Populations	EEG-Based Devices	Cardiac-Based Devices	sEMG-Based Devices	Accelerometer-Based Devices	Multimodal Devices
infancy	continuous EEG monitoring from a limited number of electrodes is routinely performed in neonatal intensive care units	sometimes used as a part of multimodal devices	limited use; monitoring muscle activity is challenging in infants	limited use; lack of consistent movement patterns during seizures	sometimes used combining EEG, PPG and movement sensors
children	useful especially in children with absence seizures	rarely used; applied mainly as a part of multimodal devices	useful for detection of generalized and bilateral tonic-clonic seizures	useful for detection of generalized and bilateral tonic-clonic seizures	increasingly used for the detection of various different seizure types
intellectual disability	useful, especially for those unable to tolerate routine EEG or inpatient video-EEG monitoring	less commonly used; applied mainly as part of multimodal devices	useful for detection of generalized and bilateral tonic-clonic seizures as well as for other major motor seizures	useful for detection of generalized and bilateral tonic-clonic seizures as well as for other major motor seizures	increasingly used for the detection of various different seizure types

**Table 3:** Wearable seizure detection devices with a CE (Conformité Européenne) mark and/or FDA approval.

Device Name	CE Mark	FDA Approval
Empatica Embrace 2	+	+
BrainSentinel SPEAC System	+	+
Epi-Care Free	+	
NightWatch	+	
Biovotion Everion	+	
ByteFlies Sensor Dots	+	
Nelli-Neuro Event Labs	+	
Neuronaute EEG System and IceCap—BioSerenity		+
CeriBell Seizure Detection Headband		+

**Table 4:** Research gaps and their possible solutions in the future.

Research Gap	Description	Possible Solutions for the Future
limited sensitivity and high false alarm rates	many devices provide limited sensitivity and especially high false alarm rates leading to unreliable detection	develop advanced algorithms incorporating machine learning to increase sensitivity and reduce false alarm rates; provide robust datasets for training these algorithms
limited detection of non-convulsive seizures	many devices are optimized for detecting tonic-clinic seizures, but have problems detecting focal seizures without major motor manifestations	integrate multi-modal sensors (EEG, ECG, motion sensors etc.) to detect a broader range of seizure types, including subtle seizures without major manifestations
battery life and power consumption	many devices require frequent recharging, limiting their long-term usability	explore low-power sensors, energy-efficient algorithms, and possibly energy harvesting technologies to extend battery life
comfort and wearability	many devices are bulky or uncomfortable resulting in poor patient adherence	improve device design focusing on lighter materials, smaller form factors and more comfortable wear options
real-time data processing and communication	many devices rely on cloud-based data processing, which can result in detection delays and data privacy concerns	develop edge computing solutions for real-time data processing on the device itself, thus minimizing dependence on cloud and improving response times
long-term monitoring and adaptation	devices usually do not adapt to long-term changes in seizure patterns or the individual patient’s condition	implement adaptive algorithms taking into account changing seizure patterns over time; develop personalized patient-specific algorithms
limited integration with healthcare systems	most devices operate in isolation and do not integrate with healthcare platforms for timely intervention	design devices that allow seamless integration with healthcare platforms using secure cloud systems, and thus facilitate remote monitoring and interventions
cost and accessibility	many devices are expensive and not easily accessible, which limits their use in larger patient populations	develop more affordable alternatives through mass production, government subsidies and open-source algorithms
user education and training	patients and caregivers may lack knowledge or skills to effectively use the devices	create user-friendly interfaces with clear instructions; offer training programs and support services
standardization and regulation	lack of universally accepted standards for wearable seizure detection devices and limited regulatory frameworks	apply the published standards for testing and validation of seizure detection devices; collaborate with regulatory bodies and standardization organizations to create clear guidelines for devices
